# The role of hospital antimicrobial and infectious diseases pharmacists in the UK: a theoretically underpinned exploration

**DOI:** 10.1093/jacamr/dlac136

**Published:** 2023-01-11

**Authors:** C Micallef, D A Enoch, P Kamranpour, R Santos, N Tyler, S Scott

**Affiliations:** Pharmacy Department, Addenbrooke’s Hospital, Cambridge University Hospitals NHS Foundation Trust, Cambridge, UK; Clinical Microbiology & Public Health Laboratory, UK Health Security Agency, Addenbrooke’s Hospital, Cambridge University Hospitals NHS Foundation Trust, Cambridge, UK; Pharmacy Department, West Hertfordshire Teaching Hospitals NHS Trust, Watford, UK; Pharmacy Department, Addenbrooke’s Hospital, Cambridge University Hospitals NHS Foundation Trust, Cambridge, UK; Pharmacy Department, Addenbrooke’s Hospital, Cambridge University Hospitals NHS Foundation Trust, Cambridge, UK; Pharmacy Department, Royal Papworth Hospital NHS Foundation Trust, Cambridge, UK; School of Pharmacy, University of East Anglia, Norwich, UK; School of Healthcare, University of Leicester, Leicester, UK

## Abstract

**Objectives:**

We sought to characterise the role of hospital infection pharmacists in the UK and to understand the core challenges being faced, future role development desires and the required support to address these.

**Methods:**

We developed a questionnaire underpinned by the theoretical domains framework exploring the barriers and enablers to pharmacists fulfilling their perceived roles and responsibilities. Any pharmacist whose role included ‘specialist antimicrobial’ or ‘infectious diseases’ was invited to complete a questionnaire sent via national infection and pharmacy groups/networks. Descriptive statistics were used to report responses to each item, and a content analysis was undertaken to summarize the key messages from an extended response option.

**Results:**

Of the 102 respondents, 91 (89.2%) were from English hospitals. Fifty-three (52%) were from district general hospitals and 45 (45.1%) from teaching hospitals. Most (97, 95%) respondents were of a senior grade. The need for a comprehensive educational programme, recognition of research as core to the role and integration with infection/microbiology departments were key requirements along with protected time to engage with the activities. Highlights of the role were opportunities to teach, making a significant contribution to patient care and scope to contribute to strategy and vision. The COVID-19 pandemic negatively impacted on respondents’ capacity to undertake their perceived roles and responsibilities.

**Conclusions:**

Our study delineates the need for UK infection and pharmacy policy makers to review hospital infection pharmacist developmental pathways and roles. Joint learning, and closer working, with infection/microbiology departments may be an efficient strategy to address the issues raised.

## Introduction

Antimicrobial resistance (AMR) represents a serious global threat and is now a leading cause of mortality globally. There were an estimated 4.95 million deaths globally associated with bacterial AMR in 2019.^1^ A report conducted by the UK Review on AMR found that AMR could be responsible for the deaths of 10 million people globally by 2050.^2^ Effective antimicrobial stewardship (AMS) strategies are paramount to curbing the spread of resistant microorganisms.^2^ Since the COVID-19 pandemic commenced, a number of reports have highlighted an increase in antimicrobial prescribing. This trend may accelerate the development and dissemination of resistant strains, thereby counteracting AMS initiatives.^3–5^

The WHO and public health guidance in a number of countries advocate for an antimicrobial pharmacist to facilitate the delivery of AMS strategies and their implementation.^6–11^

Previous UK-based research has highlighted the role of the hospital infection pharmacist (HIP) as a key member of the AMS team to support and deliver AMS initiatives, thereby optimizing patient care. The HIP’s intended role includes antimicrobial policy and guideline development, responding to complex infection-related clinical queries, monitoring antimicrobial consumption and acting as a link between microbiology and pharmacy departments.^12–16^ However, the extent to which these activities are undertaken by HIPs in practice, and whether or not they have changed or expanded in view of the COVID-19 pandemic, is unclear.

We aimed to characterize the current role of UK HIPs and also sought to explore HIP goals for their future roles and development and the support they need to achieve them. We aimed to do this within the context of the current COVID-19 pandemic.

## Methods

We undertook a cross-sectional questionnaire, underpinned by the theoretical domains framework (TDF), and invited all HIPs in the UK to respond. To develop the questionnaire, we used our combined experience, which consisted of HIPs (C.M., P.K., R.S. and N.T.), a consultant medical microbiologist (D.A.E.) and a behavioural scientist (S.S.) in addition to relevant literature^12–17^ to adapt standard TDF domain questionnaire statements^18–20^ to address the study aim.

To develop the questionnaire, we tried to characterize the role and activities of HIPs. Questionnaire items were underpinned by the TDF. Eleven questionnaire items had response options on a 5-point Likert scale, from strongly disagree to strongly agree, and an extended response option was provided for each.

The questionnaire was piloted with collaborators who were representative of the target audience, but did not participate in the study, to inform minor refinements and to establish face and content validity. The final questionnaire was hosted on Microsoft^®^ Forms, which also hosted study information, the consent form and a demographic data collection form.

### Ethics and participant identification and recruitment

Research ethics approval was obtained from the University of East Anglia Faculty of Medicine and Health Research Ethics Committee. All UK pharmacists whose role includes specialist antimicrobial or infectious diseases were invited via infection networks and organizations, using their usual methods of communication with members, including newsletters, mailing lists and social media platforms. The questionnaire was available for respondents to complete from 1 February to 31 May 2021. Reminders were sent to those who did not respond to our initial e-mail.

Entry into a prize draw for a £50 shopping voucher was offered as an incentive to participate. Respondents could provide an e-mail address if they wanted to be in the prize draw. The winner was notified by e-mail and accepted the prize. Once this was completed, we removed all e-mail addresses prior to analysing the data so it was impossible to identify any individual respondent. The winner was selected via a random number generator and sent the prize electronically.

### Data analysis

Descriptive statistics were used to report responses to questionnaire items with a Likert scale responses option, and median and IQRs were calculated for each. Content analysis was undertaken for extended response questions. One researcher (C.M.) coded extended response text inductively for barriers and enablers, which were then mapped to the relevant TDF domain(s). Mapping was checked by a behavioural scientist (S.S.) and any discrepancies resolved through a meeting and discussion. Referral to a second behavioural scientist external to the research team for adjudication was undertaken in the event of disagreement.

## Results

The questionnaire was completed by 102 HIPs, and the majority (91, 89.2%) were from England. A total of 97 (95%) identified as senior pharmacists. Nine (8.8%) identified as consultant pharmacists. Just over half (53, 52%) were from district general hospitals, with the remainder from larger teaching and tertiary hospitals. Only 22 (21.6%) of respondents declared that they dedicated 81%–100% of a full-time role to infection-related duties; see Table S1 (available as Supplementary data at *JAC-AMR* Online).

Table 1 provides summary responses to 5-point Likert questionnaire items. Over three-quarters (79.4%) agreed/strongly agreed that pharmacists should integrate within microbiology/infectious disease departments, and a similar proportion (88%) agreed/strongly agreed that they would like access to current training for speciality registrars in infection/clinical scientists. Almost half (45.1%) disagreed/strongly disagreed that they were able to provide the level of service they wanted in their roles. Although the majority of respondents (68.6%) agreed/strongly agreed that research and development was an important part of their role, most (66, 61.8%) did not feel that they have enough support to undertake these activities.

**Table 1.** Summary responses to 5-point Likert questionnaire items

Statement *Related theoretical domains framework domain*	Strongly disagree, *n* (%)	Disagree, *n* (%)	Neither agree nor disagree, *n* (%)	Agree, *n* (%)	Strongly agree, *n* (%)
I would like to see pharmacists within my speciality routinely integrated into microbiology/infectious diseases departments *Social/professional role and identity*	6 (5.9)	2 (2.0)	13 (12.7)	36 (35.3)	45 (44.1)
I would like to be able to access training programmes currently reserved for speciality registrars in infection/clinical scientists *Environmental context and resources/knowledge/skills*	4 (3.9)	0	9 (8.8)	36 (35.3)	53 (52.0)
I feel that I am able to provide the level of service I would like to within my current role *Optimism*	5 (4.9)	41 (40.2)	19 (18.6)	33 (32.4)	4 (3.9)
I feel that I have adequate career progression opportunities available to me *Environmental context and resources*	8 (7.8)	30 (29.4)	25 (24.5)	33 (32.4)	6 (5.9)
I feel that research and development is an important part of my role *Goals*	6 (5.9)	11 (10.8)	15 (14.7)	50 (49.0)	20 (19.6)
I feel that I have enough support to conduct research activities within my current role *Social influence*	19 (18.6)	44 (43.1)	20 (19.6)	18 (17.6)	1 (1.0)

Analysis of the extended responses identified key barriers and enablers mapped to the TDF. TDF domains for knowledge, social/professional role and emotion were mostly associated with enablers whereas environmental context and resources and social influence were associated with barriers. Table 2 maps each TDF domain to barriers and enablers linked to selected verbatim quotes.

**Table 2.** Extended responses and the identified barriers and enablers mapped to the theoretical domains framework

Theoretical domains framework domain	Barrier/enabler	Quote
Knowledge	Constant learning and teaching others (Enabler) Gaining and sharing knowledge (Enabler) Showcasing pharmacy role (Enabler)	I like the fact that I'm constantly learning new things and that I can pass this knowledge to the people I work with *(P16 Senior Pharmacist at a District General Hospital)* Specialist knowledge, representation of profession *(P65 Senior Pharmacist at a Teaching Hospital)*
Skills	Lack of appropriate skills (Barrier)	My lack of innovative IT skills *(P21 Senior Pharmacist at a Teaching Hospital)*
Social/professional role and identity	Improving patient care and safety (Enabler) Providing strategic leadership and vision (Enabler) Empowering healthcare professionals (Enabler)	Optimizing and improving patient care and safety is at the forefront of our trust AMS strategy *(P2, Senior Pharmacist at a District General Hospital)* To try and encourage other pharmacists to take more of an active role in antibiotic use. To try and ensure patients are well managed particularly in relation to TDM *(P3 Junior Pharmacist at a Teaching Hospital)* Simplistically, to get patients better and home. To educate others to do similar. To contribute regionally and nationally to this *(P4 Senior Pharmacist at a District General Hospital)* Making a change to improve patient care and working to improve patient safety *(P10 Senior Pharmacist at a District General Hospital)* Dynamic role, multifaceted and more than clinical bedside pharmacy. Able to influence direction of travel for the Trust and local health economy, well respected role *(P13 Senior Pharmacist at a District General Hospital)* Provide strategic support on antimicrobial stewardship within our organization in line with the national strategy *(P20 Senior Pharmacist at a Teaching Hospital)* Empowering nursing staff, education and training, autonomy, work with IPC *(P22 Senior Pharmacist at a Community Hospital)* Improve AMS with a view to educate all members of the healthcare team to ensure this responsibility is embedded in their day-to-day clinical practice *(P69 Senior Pharmacist at a Teaching Hospital)* Ensuring safe and moreover effective antibiotic treatment for patients, improving their outcomes. Pride in our stewardship work *(P74 Senior Pharmacist at a District General Hospital)* The difference I make in patient care and the PR my profession gets *(P91 Senior Pharmacist at a District General Hospital)*
Goals	Competing priorities (Barrier) Trust Board engagement (Barrier) Making a significant difference (Enabler) National engagement (Enabler)	Providing the best care for patients and optimizing their antimicrobial therapy. Engagement in AMS from senior clinicians and Trust management—this can make it difficult to get buy-in and engagement from consultants and therefore junior staff *(P7 Senior Pharmacist at a District General Hospital)* Support from Trust Board *(P19 Senior Pharmacist at a District General Hospital)* Hospital Boards understanding AMS is different but connected to IPC and the disparity in funding for staff between the 2 *(P36 Senior Pharmacist at a Teaching Hospital)* Quality improvement. Data improvement. *(P67 Senior Pharmacist at a Teaching Hospital)* AMS services do not appear to be high on the agenda locally—or at least funding for posts to achieve goals. Hoping new UK structure with national and regional leads will contribute to the profile *(P72 Senior Pharmacist at a District General Hospital)* AMS/AMR is my only priority but one of 100s for clinicians we need to enact change. Competing with other priorities is a barrier to change *(P89 Senior Pharmacist at a District General Hospital)*
Reinforcement	Feels good to help (Enabler)	Sense of reward in optimizing a patient's antimicrobial treatment to ensure that it is both effective and safe *(P24 Senior Pharmacist at a District General Hospital)*
Environmental context and resources	Lack of funding (Barrier) Lack of resources (Barrier) Lack of developmental opportunities (Barrier) Time limitations (Barrier) Hierarchy issues (Barrier) Lack of flexible working (Barrier) Lack of senior support (Barrier) Geographical differences between sites (Barrier) Better technology (Enabler) Integrated health economy and support and autonomy (Enabler)	To have more teaching and webinars to improve my clinical knowledge and maybe have access to microbiology training that are usually reserved for registrars *(P6 Senior Pharmacist at a District General Hospital)* Time, resource, support *(P31 Senior Pharmacist at a Teaching Hospital)* Standardized training—develop general microbiology knowledge but also better [antibiotic] pharmacist knowledge *(P39 Senior Pharmacist at a District General Hospital)* I am very lucky. Having support from pharmacy, IPC and ID/micro to undertake activities and autonomy. Also integrated across the health economy *(P56 Senior Pharmacist at a District General Hospital)* Working from home/micro dept for abx time so I am not interrupted by pharmacy colleagues *(P66 Senior Pharmacist at a District General Hospital)* Insufficient pharmacist staffing *(P70 Senior Pharmacist at a District General Hospital)* Integration with the micro/ID team; time constraints *(P71 Senior Pharmacist at a Teaching Hospital)* A better EPMA reporting system, regular microbiology 1:1 time, extra staffing *(P75 Senior Pharmacist at a District General Hospital)* I feel that my knowledge is insufficient for additional activities I wish to perform such as blood culture reviews, ward rounds. I do not have access to the microbiology system to view results *(P77 Senior Pharmacist at a Teaching Hospital)* Time, support from pharmacy and microbiology colleagues, support from the Trust leadership *(P79 Senior Pharmacist at a District General Hospital)* Current trust very diverse—geographically and patient cohort wise. This means it is difficult to visit each area and engage with staff and patients *(P82 Senior Pharmacist at a Community Hospital)*
Social influence	Conflicts between pharmacy and microbiology departments (Barrier) Lack of engagement from medical leadership and engagement (Barrier) Lack of professional acknowledgement (Barrier)	Differing opinions of consultant microbiologists can be one of the biggest obstacles to overcome. COVID-19 has led to staff shortages *(P2 Senior Pharmacist at a District General Hospital)* Conflict between microbiology lead and pharmacist boss, i.e. not enough role definition *(P14 Senior Pharmacist at a District General Hospital)* Resources, time, strong medical leadership across all specialties, engagement from frontline staff *(P17 Senior Pharmacist at a District General Hospital)* Staffing pressures, clinical ward work. Increased support from senior management and recognition of the value in the role *(P40 Senior Pharmacist at a District General Hospital)* Lack of top-level management support for the antimicrobial pharmacist role *(P59 Senior Pharmacist at a District General Hospital)* Better understanding and engagement from senior medics and nursing colleagues to support our interventions in certain specialties *(P83 Senior Pharmacist at a District General Hospital)*
Emotion	Passion (Enabler) Desire for positive change (Enabler)	I am a very passionate pharmacist and I strongly believe that right interventions could lead to a positive change *(P25 Senior Pharmacist at a District General Hospital)* I'm passionate about improving antimicrobial stewardship and patient care and safety. This is really what drives me on a daily basis *(P59 Senior Pharmacist at a Teaching Hospital)*

EPMA, electronic prescribing and medicines administration; ID, infectious diseases; IPC, infection prevention and control; TDM, therapeutic drug monitoring.

## Impact of COVID-19

We asked whether COVID-19 had impacted on the professional and personal lives. The main themes which emerged from the content analysis of this question highlighted the increase in workload to HIPs, especially the increased pharmacy (non-infection-related) duties. In addition, many felt that they were not able to perform their routine AMS duties. Some also mentioned the physical and mental impact the pandemic was having on their lives and on the ability to perform their duties.

A snapshot of responses is provided below (see Supplementary data for the full set of responses).

‘it has completely taken over my job—every aspect’

P12 Senior Pharmacist at a Teaching Hospital

*‘*…affected how I conduct my prescribing clinics, more telephone appointments which make it quite difficult to reach out to complex patients’

P18 Senior Pharmacist at a Teaching Hospital

*‘*pulled to provide non-AMS duties, including ward cover, limiting the amount of time to undertake AMS activities’

P69 Senior Pharmacist at a Teaching Hospital

*‘*Both- long COVID fatigue. Having to cover wards. I am now based on Acute COVID ward…’

P35 Senior Pharmacist at a District General Hospital

*‘*it has been difficult to stay motivated in any aspect of work when life outside of work is so restricted and depressing…’

P41 Senior Pharmacist at a District General Hospital

*‘*Personally I’ve become very mentally unwell from the unrelenting workplace pressure/stress’

P66 Senior Pharmacist at a District General Hospital

## Discussion

To our knowledge, this is the first study that used TDF to explore HIP current roles and future development and training needs in the UK. TDF domains for knowledge, social professional role, goals and reinforcement largely comprised enablers. HIPs were keen to showcase the pharmacy role in AMS, enjoyed learning and teaching others and empowering healthcare professionals. HIPs had a direct impact on improving patient care and wanted to make a difference.

The main barriers were found in the social influence and environmental context and resources TDF domains. Lack of funding and time were the main barriers in the environmental context and resources domain, and lack of engagement from senior managers and conflicts between pharmacy and infection departments were the main barriers in the social influence domain.

HIPs stated that they were unable to deliver the AMS services they wished and felt that they needed more support from senior hospital leadership. Some felt that they did not have adequate training to conduct their role effectively and wanted access to specialist training accessible to medical/scientific colleagues. The COVID-19 pandemic impacted negatively on service delivery and wellbeing of our respondents. This is consistent with a Royal Pharmaceutical Society survey, which found that nearly 90% of pharmacists were at risk of burnout.^21^

Interestingly, constant learning and teaching others was an enabler in the knowledge domain, but one of the barriers in environmental context and resources was that organizations do not provide sufficient opportunities to develop knowledge.

Limitations to the study include its UK bias; HIPs may have different roles and responsibilities across the world, which are not reflected here.

### Conclusions

Our study has important implications for UK policy makers in the fields of infection and pharmacy. A comprehensive infection-training programme, which is already available to speciality registrars and clinical scientists, should be expanded to include infection pharmacists as a matter of priority. Hospital infection pharmacists need to be fully integrated within the wider multidisciplinary infection teams/departments, with clearly defined roles and responsibilities and not used to provide general pharmacy cover, as this greatly restricts the impact of their AMS activities, which in turn affects their ability to deliver on national AMR/AMS targets and direct patient care. National UK AMR/AMS leads and senior pharmacy managers must work with hospital chief pharmacists and pharmacy leaders to review job descriptions to fully integrate an AMS practice-based research element. Further qualitative research on this topic is urgently warranted.

## Supplementary Material

dlac136_Supplementary_Data

## Transparency declarations

This work has been presented in part at the FIS 2021 conference and won best poster award (2nd place). C.M. has consulted for Pfizer and Mundipharma Research Ltd. C.M. received an educational grant from Pfizer. D.A.E. has consulted for Pfizer, MSD, Tillots Pharma UK Ltd and Shionogi. N.T. received consultancy fees to deliver talks and/or attend conferences by Pfizer, Tillots Pharma UK Ltd, Gilead, Guidepoint Global Advisors and Researchpartnership.com. R.S., P.K. and S.S. report no conflicts of interest.

## Supplementary data

Table S1 is available as Supplementary data at *JAC-AMR* online.
