# Supplementary material for: The role of hospital antimicrobial and infectious diseases pharmacists in the UK: a theoretically underpinned exploration
Source: JAC Antimicrob Resist. 2023 Jan 11;5(1):dlac136. doi: 10.1093/jacamr/dlac136 (PMC9833282; doi:10.1093/jacamr/dlac136)
Supplement: dlac136_Supplementary_Data [file dlac136_supplementary_data.docx]

**Supplementary Table 1 Hospital infection pharmacist respondent characteristics**

| **HIP Characteristics** | **Number (%)** |
| --- | --- |
| Country |  |
| England | 91 (89.2) |
| Scotland | 4 (3.9) |
| Wales | 7 (6.9) |
|  |  |
| Agenda for change banding* |  |
| 7 (junior pharmacist) | 4 (3.9) |
| 8a (senior pharmacist) | 57 (55.9) |
| 8b | 29 ( 28.4) |
| 8c | 10 (9.8) |
| 8d | 1 (1) |
| Prefer not to say | 1 (1) |
|  |  |
| Consultant pharmacist | 9 (8.8) |
|  |  |
|  |  |
| Organisation |  |
| Community hospital | 2 (2) |
| District general hospital | 53 (52) |
| Teaching hospital | 45 (45.1) |
|  |  |
| Proportion of time spent on infection pharmacist role |  |
| 1-20% FTE | 7 (6.9) |
| 21-40% FTE | 20 (19.6) |
| 41-60% FTE | 27 (26.5) |
| 61-80% FTE | 26 (25.5) |
| 81-100% FTE | 22 (21.6) |

*AFC: Agenda for Change is a UK National Health Service salary scheme where Band 8 a-d are considered senior managers and AFC 8d is considered most senior. Consultant Pharmacists are considered senior pharmacists and usually are AFC 8b and above.

FTE: Full time equivalent
